# Pancreatic Transplantation, the 8th Marjorie Budd Lecture

**Published:** 1989-02

**Authors:** Peter Morris, M. G. Wilson

**Affiliations:** Nuffield Professor of Surgery, University of Oxford, 4th February 1989 at Bristol Royal Infirmary


### Pancreatic Transplantation 8th Marjorie Budd Lecture

**Delivered by Professor Peter Morris**
Nuffield Professor of Surgery, University of Oxford, 4th February 1989 at Bristol Royal Infirmary.

Though the introduction of insulin in 1922 immediately transformed the management of diabetes, allowing diabetics to survive with their disease, the microvascular complications of the disease became increasingly evident with time. Thus insulin-dependent or type 1 diabetes is the fifth major cause of renal failure, the major cause of blindness, and coronary heart disease is a major cause of death in these patients. Evidently insulin is not the whole answer to diabetes and it was hoped that pancreatic transplantation might provide normal control of glucose levels throughout a 24 hour period, and so completely correct the metabolic abnormality in the diabetic patient which, in turn, might cause regression or failure of progression of these microvascular complications of disease.

Lillehei, in 1966, was an early pioneer in pancreatic transplantation; most of his attempts failed but he did have one patient who survived for one year completely insulin-independent before being killed in a car accident. Since the advent of cyclosporin pancreatic transplantation has 'taken off'; the average 1 year success rate is 40%, but some units have achieved a 70–80% one year graft survival rate recently.

However pancreatic transplantation, whether segmental or whole organ, is a major operation with a 30% complication rate and an average hospital stay of 6 weeks. Moreover, it has not been shown that either neuropathy or retinopathy regresses probably because surgery at present is done at an end-stage of the disease. If transplantation of pancreatic endocrine tissue is to succeed it should be done at an early stage, but such a hazardous operation cannot be offered to diabetics soon after their onset of their disease, or soon after the first evidence of microvascular complications. Thus it looks as though it will never be an option except in late cases who also need a renal transplant and hence immunosuppression.

The search is now on to find a means of transplanting isolated pancreatic islets by a simple safe, non-operative, injection procedure. A successful method of isolating islets was developed in rats which gave rise to early optimism. Rats made diabetic by streptozotocin (a $\beta$ cell toxin) can be cured by injection of a suspension of their own islets into their portal vein, spleen or beneath the kidney capsule and 'live happily ever afterwards'. Allografts in rats, however, are rejected promptly and it has proved difficult to achieve prolonged survival with a variety of immunosuppressive protocols, including cyclosporin, but progress is being made to overcome this problem.

However, the techniques used in the rat for the isolation of islets was unsatisfactory for large animals or man. The development of a technique in Oxford for the preparation of islets from a human pancreas using collagenase at 39°, injected directly into the pancreatic duct, has allowed good yields of human islets to be obtained. The monkey has proved a good preclinical model for testing the technique in that the pancreas resembles very closely that of man and it is possible to prepare islets using the same technique and implant them in totally pancreatectomised recipients and show that autologous grafts will survive for up to 3 years.

Cautious clinical trials are planned in the near future but there are many problems still to be overcome. If a safe successful technique can be found the supply of islets from cadaver pancreas will never meet the demand. Suspensions must be used fresh but may be kept in culture for a limited time, perhaps up to 7 days. There is still so much to learn about the physiology of normal islets as well as diabetes. For example, we do not even yet know why the islets are situated in the pancreas, perhaps the surrounding exocrine pancreatic cells have some important relationship with the islets. We must hope not!

This lecture was a fascinating insight into the important research in the treatment of a common and sometimes devastating disease. It was particularly appropriate as an update on the 4th Marjorie Budd Lecture given in 1985 by Sir Roy Calne on 'Liver and Pancreas transplantation'.

M. G. Wilson

